# Testing the visual field of children and adults with Rarebit: The role of task repetition on sensitivity

**DOI:** 10.1371/journal.pone.0221122

**Published:** 2019-08-13

**Authors:** Iryna Tachyla, Luca Battaglini, Michele Barollo, Simone Cosentino, Giulio Contemori, Luisa Pinello, Ambra Ciavarelli, Clara Casco

**Affiliations:** 1 Pediatric Low Vision Center, Department of Pediatrics, University of Padua, Padua, Italy; 2 Department of General Psychology, University of Padova, Padova, Italy; 3 Neuro.Vis.U.S. Laboratory, University of Padova, Padova, Italy; Justus Liebig Universitat Giessen, GERMANY

## Abstract

Rarebit is a simple and user-friendly perimetry that tests the visual field by using tiny supra-threshold dot stimuli. It appears to be especially useful for examining the visual field of children who are under 12 years of age. However, previous data showed that the number of errors was higher in children than adults. We ask whether the different number of errors in these two groups depended on task learning and whether it may be accounted for by sensitivity differences or a response bias. Thirty-one children between 9 and 12 years of age and thirty-nine adults were tested three times with Rarebit perimetry. A bias-free sensitivity index, *d’*, rather than the simple hit rate, revealed a group difference that remained after extensive task repetition. Indeed, *d’* increased with task learning in a similar way in the two groups so that group difference remained after practice. The response bias differed in the two groups, being conservative in the older group (criterion *C* >0) and liberal in the younger (criterion *C* < 0). Both biases disappeared with task learning in the third session, suggesting that response bias cannot account for the group difference in sensitivity after practice. When bias-free measures of sensitivity are used and task learning effects are minimized, Rarebit perimetry may be a more valuable method than simple mean hit rate (MHR) to enlighten sensitivity differences in the visual field assessment within the pediatric population.

## Introduction

Visual field examination is routinely performed in adult populations by measuring sensitivity to visual stimuli using computerized static perimetry. Many factors, such as practice effects, visual uncertainty, gaze fixation maintenance and verbal instructions may affect sensitivity measurements [1–3]. The decisional criterion (i.e., the capability to select the appropriate response for the perceived stimulus and for inhibiting irrelevant responses) is particularly sensitive to the way verbal instructions are given and may induce either liberal or conservative behavior [1]. Visual field examination is also often used in the pediatric population [4–13]. Most of these studies found that adult performance levels are achieved by 12 years of age. In younger children, performance often diminishes when visual functions are tested. Non-sensory factors contribute more to reduced performance in children than in adults. Difficulties in task learning [8,11,14] in maintaining a stable fixation on the central target [7] and in sustaining attention, vigilance and concentration [2,9,11,14] are commonly reported and may affect visual field evaluation in children.

One way to obtain reliable data when measuring the visual field in children is to use suprathreshold (highly discriminable) stimuli. Frisén [15] proposed a perimetric method, Rarebit perimetry, which uses small, very intense target dots (microdots or “rare bits”). By probing the detection of target dots within each of a set number of rectangular areas, Rarebit allows for the detection of tiny distributed areas of absolute blindness within otherwise normal areas of vision. The use of these suprathreshold stimuli is suitable because they provide hit rates that do not vary with eccentricity in normal eyes. Indeed, Rarebit perimetry returns, in trained adults, hit rates close to 100% at all eccentricities. In addition, spatial uncertainty about the location of low-intensity targets is reduced when using high contrast, highly discriminable stimuli [16]. Moreover, the difficulty in maintaining a stable fixation on the central target [1,7,17–18], which may strongly affect the testing of peripheral visual sensitivity, is reduced by the use of a moving fixation mark and a short stimulus duration (200 ms) that is shorter than the average latency for a re-fixation movement.

Despite these advantages, children’s performance in Rarebit perimetry is reduced compared to that of adults. Martin [7] showed that only 76% of children between 6.5 and 12 years of age showed reliable results when mean hit rate (MHR) and errors were considered, compared to 90% of adults. Although reliable visual field examinations obtained with Rarebit were higher than those obtained with the frequency-doubling perimetry (76% vs. 57% of children and 90–95% vs. 90% of young adults, respectively), median MHR was significantly lower in children than in adults. Moreover, there was a significant correlation between age and Rarebit errors.

Although Rarebit perimetry has been shown to be more appropriate than standard automatic perimetry for testing the visual field of children [2], several factors may account for children’s lower performance compared to that of adults. Because Rarebit involves random fixation changes, spatial attention must be shifted continuously in order to perform accurate saccades towards each new random location. Fixation inaccuracy due to location uncertainty and attention lapse may increase the probability of wrong responses: either the observer does not respond to the target when it is present (misses) or responds when the target is absent (false alarms, FA). Errors might be reduced by task learning, as established by several studies [19–22]. Regarding Rarebit, however, the role of task learning remains unclear. One study observed that task learning affected error rates [22] whereas a second study [21] did not find a significant effect of learning. Neither study distinguished the effect of misses (1-MHR) from that of FA, both of which are Rarebit outcomes.

Both misses and FA can be used to calculate two independent parameters, according to Signal Detection Theory (SDT) [23–26]. One such parameter is *d’*, a bias-free parameter that may produce sensitivity values different from those obtained with the standard MHR used in most studies [7,12]. The other is the criterion (C), by which the observer chooses a location along the internal response axis and responds either “yes” whenever the internal response is greater than C or “no” whenever the internal response is less. Importantly, C may change while *d’* remains unvaried. With no response bias, C reflects equal probability of misses and FA. A biased criterion can result in responses from participants that range from very liberal (resulting in more FA than misses) to very conservative (with more misses than FA).

Distinguishing sensitivity from response bias in accounting for age differences is not easy. For instance, Tschopp and colleagues found evidence of conservative behavior in children who were tested using Octopus perimetry [13], with misses larger than FA, but whether changes in C affected the sensitivity measurement remained unclear.

However, psychophysical literature has shown that sensitivity and response bias can be distinguished and are affected differently by practice [27]. In particular, Gold and Ding [28] discussed the effects of internal, external and extraneous noise on *d’* and C. Signal strength and noise either internal to the neural system or external (environmental) affect stimulus representation, indexed by *d’*. This is particular relevant when assessing visual field in clinical population. Indeed, it has been proposed, that most retinal diseases impair visual function by increasing the level of noise within the visual pathway [29].

Motivation and rewards selectively affect C. Other non-sensory factors (extraneous noise), including spatial uncertainty, lapse of attention and insufficient task learning, have been proven not only to increase response bias but to reduce sensitivity even in the presence of strong signals.

The present study measured two sensitivity indices (MHR and *d’*) and response bias (as measured by its criterion, C) to establish how these affected visual field assessments in adults and children using the Rarebit technique. We also investigated whether the effects of age upon these parameters depend on task learning.

## Method

### Participants

One group of 39 adults ranging from 20 to 32 years of age (average = 27.2, st. dev = 6.1, 20 females and 18 males) and 31 children ranging from 8.5 to 12 years of age (average = 9.9, st. dev = 1.2, 16 females and 15 males) participated in our study. To recruit participants, we referred to friends and colleagues that could either personally participate in the study or enquire whether the children of an acquaintance of them were willing to participate. All participants had a best corrected visual acuity (VA) of at least 1.0 (20/20), full visual field and absence of ocular/neuro-ophthalmological disease. None of the participants was identified by teachers (as required by a specific Italian law; Law 170, October 2010) or parents as having a diagnosis of dyslexia, learning disabilities or diseases that may cause any loss of visual field sensitivity. The participants participated voluntarily without any compensation, and we obtained oral consent from all participants and written consent from the parents of the children prior to their inclusion in the study. The investigation was conducted in accordance with the Declaration of Helsinki of 1975 (as revised in Tokyo in 2004) and received ethical approval from the University of Padova (protocol 2177).

### Apparatus and stimuli

**Fig 1:**
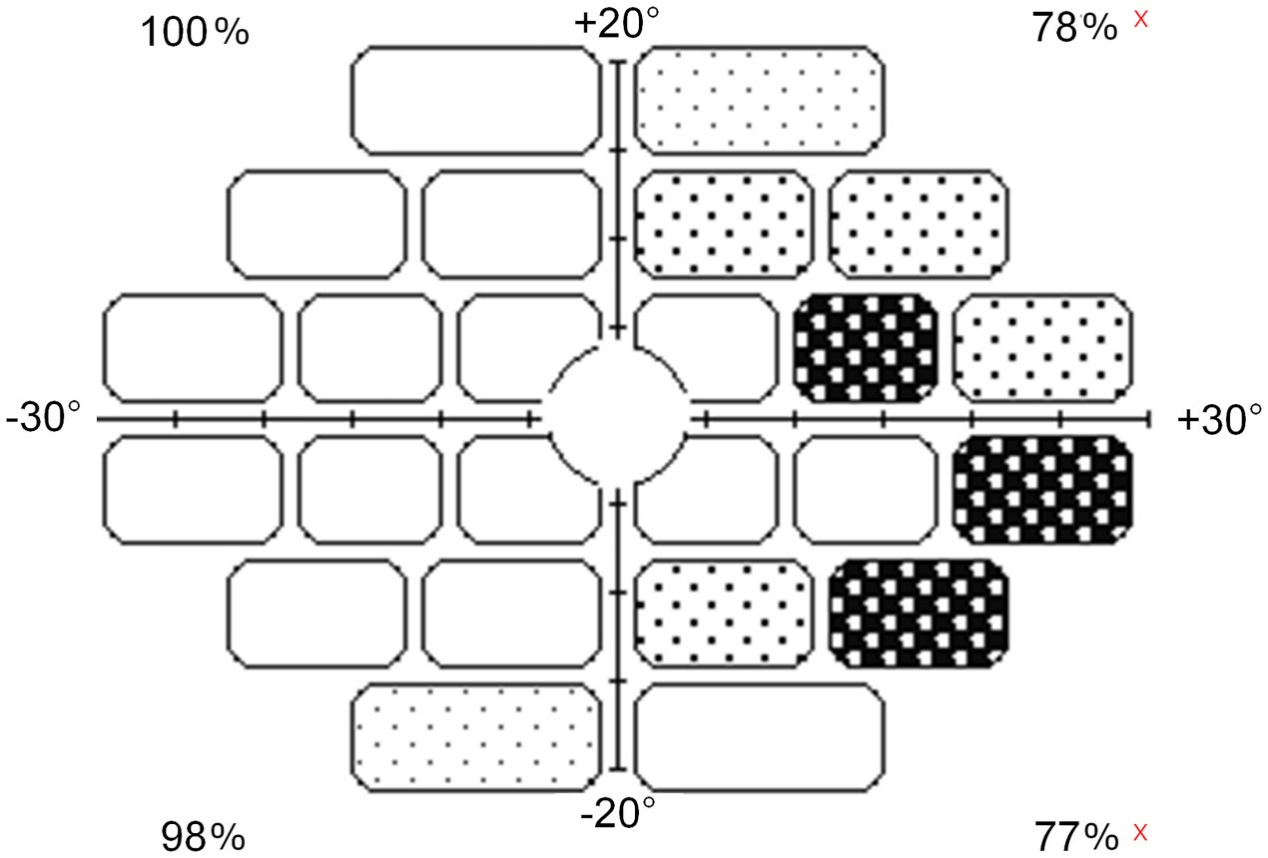
Example of outcome from Rarebit perimetry. A white rectangle indicates that all dots are perceived. The shading represents a larger proportion of non-perceived dots in that particular location (see manual for more details). Numerical results are compared to normal limits. Results falling outside limits are marked with a x next to the percentage numbers. The outcome reported in Fig 1 represents a partial scotoma in half of the visual field in a patient (non-participant in this study) suffering from homonymous hemianopia.

Following calibration, stimuli were displayed on a 19-inch CTX CRT Trinitron monitor with a refresh rate of 60 Hz. The screen resolution was 1280 x 1024 pixels, the size of the display was 40 x 30 cm and the viewing distance was 50 cm for the peripheral test and 100 cm for the central test. Each pixel subtended vertically ~1 arcmin. The mean luminance, measured using a Minolta LS-100 photometer, was 0.2 cd/m^2^ for the background and 127 cd/m^2^ for the fixation and the target stimulus. It has previously been shown that stimuli with similar luminance parameters remain suprathreshold across the visual field of young participants [22]. In the current study, Rarebit Version 4.0 was used. The test consisted of the brief (12 frames) presentation of one or two high-contrast, minuscule (about .5 of normal minimum angle of resolution [MAR] at every visual field location) light dots (microdots) against a dark background. When switching from central to peripheral testing, the size of the dot was automatically scaled with the change in viewing distance according to normal visual acuity. Pairs of dots were separated by 4 deg of visual angle (center to center) so that the two areas were tested simultaneously. Using the mouse, the participants responded with two, one or no click, depending on whether they perceived two, one or no dot. Stimuli were presented in 24 separate rectangular test areas: four central (6 x 8 degs) and 20 peripheral (6 x 14 deg). The tested visual field covered a horizontal eccentricity of 27.5° and a vertical eccentricity of 20° upwards and of 22.5° downwards. The foveal area of 4° radius (included in the ‘Foveal’ routine of the Rarebit) was not tested. Such distribution is the same for both the left and the right eye. Five stimulus repetitions for each area tested are recommended as a compromise between sufficient data collection and test time [15,30]. A total of 10% of the presentations contained only one dot or none at all. Unlike conventional perimetry, which returns the threshold (expressed in decibel), Rarebit perimetry returns a hit/miss rate (Fig 1); the lower the mean hit rates (MHRs), the larger the degree of visual field loss.

We strictly followed standard Rarebit procedure in order to avoid bias estimates due to the instructions given by the experimenter. All participants were instructed to maintain fixation on the black dot at the center of the perimeter and to press the mouse key after the stimulus became visible. They were given the following oral instructions: “Always look at the white cross. One or two small bright dots or no dots will appear. Using the mouse, respond with two, one or no click, depending on whether you perceive two, one or no dot”. After the initial demo and practice session all participants understood the task and could respond to the stimuli in the appropriate manner.

Observers sat in a dark room at the distance from the screen required for inner (central) and outer (peripheral) testing. When appropriate, corrective lenses were adjusted to accommodate for the distance. Viewing was monocular (the dominant eye was tested with appropriate correction when needed). All of the participants performed three sessions, separated by an interval ranging between two and four days. They were given initial training by presenting one stimulus in each area to become familiarized with the stimuli and the task.

### Data analysis

We used the proportion of the overall MHR (i.e., the correct responses when only two dots were presented) and the number of FA (i.e., trials in which observers reported that they perceived two dots when one or no dots were presented). We also calculated *d’* and C from the transformation of the proportion MHR and FA in z-scores [31]; *d’* is the difference between the z-transforms of MHR and FA, where the z indicates how many standard deviations a score is from the mean (z = (X-μ)/σ).

$$d^{\prime }=[z(Hits)-z(FA)]$$

The criterion C was found by averaging the z-score that corresponds to the hit rate and the z-score that corresponds to the FA rate, then multiplying the result by minus .5.

C=-0.5[z(Hits)+z(FA)]

Infinite values were avoided by adding the appropriate correction factor $\left[\frac{1}{2N}\right]$ to proportions of zero and subtracting it from the proportion of one [31]. A C value not differing from 0 reflects no bias, whereas a C value less or greater than 0 indicates liberal and conservative criterion, respectively. Unfortunately, Rarebit procedure does not distinguish the “two-dots” response when either one dot or no dots are presented. Therefore, we could not calculate *d’* separately for the two non-target conditions. Likely, the sensitivity might have been lower when the non-target was one-dot, given that the Gaussian distribution referring to the one-dot is shifted rightwards with respect to the distribution referring to no-dot (while that referring to two-dots remain fixed), thus resulting in a lower *d’*.

To evaluate the effect of age (adults versus children) and task learning (sessions 1, 2 and 3), we first checked, using a Shapiro-Wilk normality test, whether the sample distributions of MHR, *d’* and C were normal. Since the assumption of normality was justified only for C data, we analyzed all data using a Nonparametric Analysis of Longitudinal Data (nparLD) [32], with the group as the between-subjects factor and session as the within-subject factor. The Wilcoxon signed-rank test with continuity correction was used for pairwise comparisons between groups at each of the three blocks. This lead to a total of nine comparisons for *d’* and for C. To account for multiple comparison, both pairwise and zero effect comparisons have been corrected using the False Discovery Rate (FDR) method.

## Results

**Fig 2:**
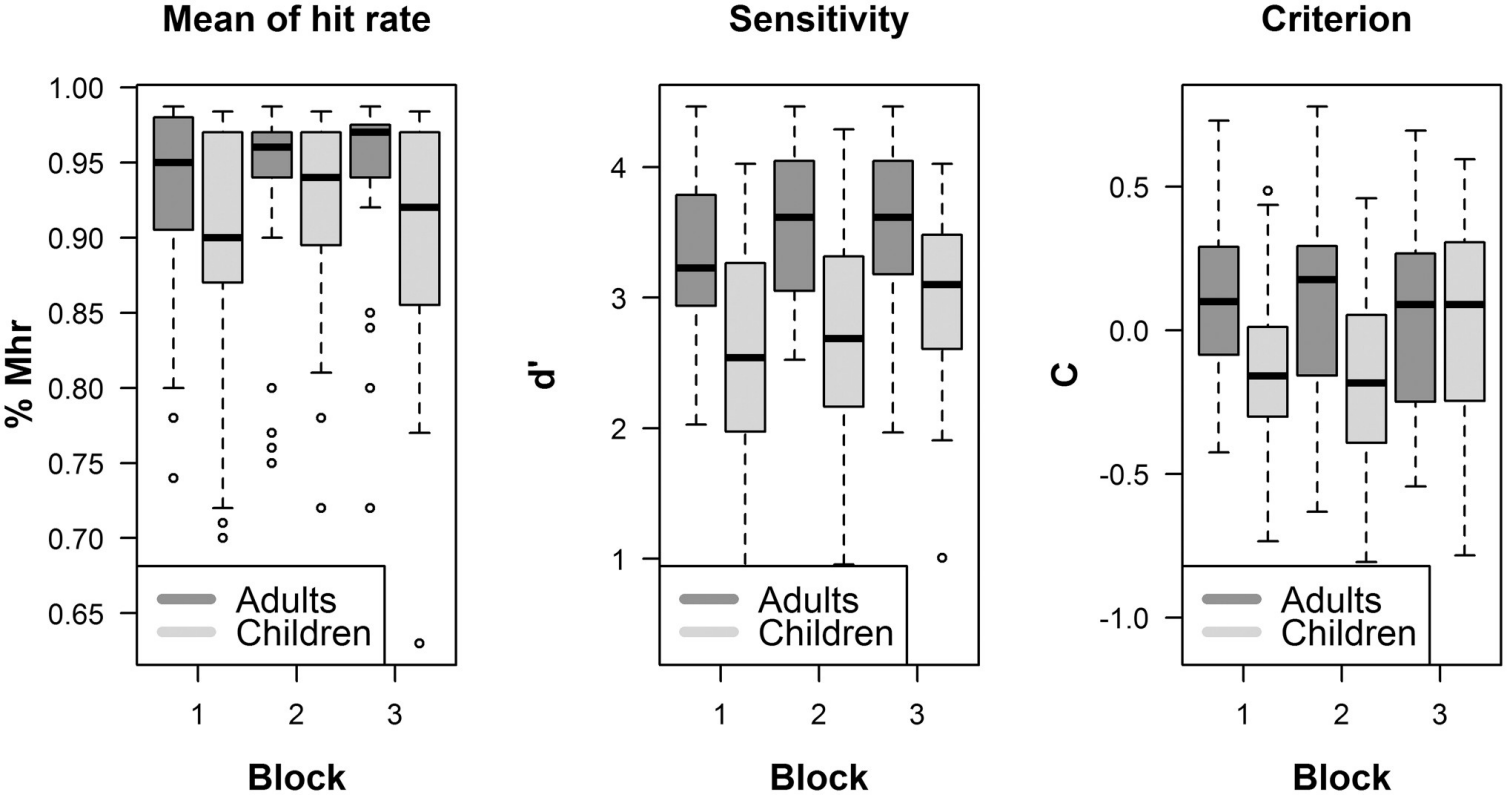
Experiment results. The Graph shows mean hit rate (MHR) (left panel), *d’* (central panel) and the criterion C (right panel) in the three sessions by the two groups. Box plots represent the minimum, first quartile, median, third quartile and the maximum distance measured for each group in each session. Dark gray represents adults’ data, whereas light gray represents children’s data.

The effects of age and task learning on MHR, *d’* and C are shown in Fig 2. The results of nparLD analysis are summarized in Table 1. The overall difference in MHR across groups is small but significant [median adults: .96; median children: .92; *p* = .008] On the other hand, neither the effect of session nor the group x session interaction resulted in a significant difference. As Fig 2 shows (left panel), there is a high number of values outside 1.5 times the interquartile range below the lower quartile in the MHR data of both groups, reflecting large individual variations.

**Table 1:** 

MHR				*d'*				C			
**Factor**	**Stat**	**df**	**Sig.**	**Factor**	**Stat**	**df**	**Sig.**	**Factor**	**Stat**	**df**	**Sig.**
Session	1.23	1.92	.29	Session	5.8	1.94	< .001	Session	.94	1.945	.39
Group	7.07	1	.008	Group	25.8	1	< .001	Group	8.6	1	.003
Group:Session	1.98	1.92	.14	Group:Session	.69	1.94	.5	Group:Session	3.8	1.945	.025
**Factor**	**Rank Means**	**Nobs**	**RTE**	**Factor**	**Rank Means**	**Nobs**	**RTE**	**Factor**	**Rank Means**	**Nobs**	**RTE**
**group**				**group**				**group**			
ADULTS	120.26	117	.57	ADULTS	128.76	117	.61	ADULTS	118.59	117	.56
CHILDREN	86.94	93	.41	CHILDREN	76.24	93	.36	CHILDREN	89.03	93	.42
**Session**				**Session**				**Session**			
1	98.57	70	.47	1	89.17	70	.42	1	100.91	70	.48
2	107.23	70	.51	2	105.04	70	.5	2	99.9	70	.47
3	104.99	70	.5	3	113.28	70	.54	3	110.62	70	.52
**group × session**				**group × session**				**group × session**			
ADULTS: 1	111.81	39	.53	ADULTS: 1	113.49	39	.54	ADULTS: 1	122.73	39	.58
ADULTS: 2	120.73	39	.57	ADULTS: 2	136.15	39	.65	ADULTS: 2	121.31	39	.58
ADULTS: 3	128.23	39	.61	ADULTS: 3	136.64	39	.65	ADULTS: 3	111.73	39	.53
CHILDREN:1	85.34	31	.4	CHILDREN:1	64.85	31	.31	CHILDREN:1	79.1	31	.37
CHILDREN:2	93.73	31	.44	CHILDREN:2	73.94	31	.35	CHILDREN:2	78.48	31	.37
CHILDREN:3	81.74	31	.39	CHILDREN:3	89.92	31	.43	CHILDREN:3	109.52	31	.52

The upper panel of the table shows the results of Nonparametric Analysis of Longitudinal Data (nparLD). For each dependent variable (mean hit rate: MHR, *d’* and criterion C) the table reports: statistics value (Stat), degrees of freedom (df), significance value (Sig.). In the lower panel Rank Means (rank mean of overall ranks), Number of observation (Nobs) without counting the repeated measurements within the cell, and the relative treatment effect (RTE, that ca be considered as a non-parametric equivalent for the effect size) for each factor level combination is reported. A RTE value of 0.5 indicates no effect. A RTE < 0.5 (or > 0.5) means a tendency for subjects in a subgroup to score lower (or higher) than a randomly drawn subject from the whole sample. The lower (or higher) the RTE, the lower (or higher) the probability [32].

The analysis of *d’* data revealed a significant effect of groups [median *d’* adults: 3.44, median *d’* children: 2.82; *p* < .001], sessions (*p* < .001) but not of the group x session interaction (*p* = .5), that is the groups differed significantly in all sessions and the difference between the first and third sessions reached significance for both groups (adults: *p* = .014; children: *p* = .005).

Criterion C results showed a significant difference across groups [median C adults: .1, median C children: -.14; *p* < .003] and a significant group x session interaction (*p* < .025), indicating that the two groups differed in the first (*p* = .001, median adults: .1, median children: -.18) and second session (*p* = .006, median adults: .17, median children: -.16), but not in the third session (*p* = .89, mean adults: .09, mean children: .08).

The C difference in the first and second sessions suggests that the children’s responses were liberal, but the adults’ were conservative. This difference in the C disappears in the third session. Indeed, the value of C did not significantly differ from zero at the one-sample Wilcoxon test (children: *p* = .79; adults: *p* = .18).

## Discussion

Rarebit is now extensively used for evaluating visual function during age span. In children, it has been used for visual field testing of normally sighted children [4–13] and children with visual deficits [33–34]. In adults, it was demonstrated useful for a visual field evaluation of patients with optic nerve or visual pathway lesions [35–36], glaucoma [37], hemianopia [38–39], macular degeneration [40], cataract [41], diabetes [42], and decline in foveal function with age, reflecting the loss of neural detectors [43]. In the present study, we aimed to disentangle sensitivity from the response bias in the performance of children and adults during visual field testing using Rarebit perimetry. In addition, we sought to establish how sensitivity and bias changed in both children and adults as a consequence of task learning.

An overall group effect was found with both MHR and with a bias-free parameter (*d’)*; only *d’* revealed that the group difference was independent of task learning, as it was significant in all sessions. Moreover, task learning increased *d’*, but not MHR. However, task learning reduced the response bias in both groups so that the group difference in C disappeared with repetition.

We find that, in visual field assessments using suprathreshold stimulation, a difference in sensitivity between children and adults is still present when the response bias is minimized and practice with the task is high. In the third session, *d’* increased in both groups, and the bias was negligible and not differing amongst adult and children groups. These effects of task repetition should seriously be taken into consideration when using Rarebit for the assessment of visual field integrity, especially in children. In fact, clinicians might benefit from repeating the test multiple times to account for learning effects.

The importance of evaluating the bias is highlighted by the finding that Rarebit testing resulted in a conservative response in adults and liberal response in children. Other studies [13] have identified differences in bias between adults and children based on FA differences. Using a different type of perimetry (Octopus 2000R), it was found that that children responses were conservative. Nilsson and colleagues [44] instead found no difference in FA between groups of children aged between 6 and 10 years tested with Rarebit. This finding indicates that the bias rate depends on the group tested and the type of visual field assessment. However, it should be remarked that using FA as an index of bias is not always appropriate because a decrease of FA indicates a conservative response if FA remain less than misses but a liberal response if they are more than misses. Therefore, C rather than FA should be used to ascertain bias in clinical practice. If these methodological guidelines were taken into consideration, Rarebit perimetry could provide a very useful instrument, to be coupled with standard visual acuity measurement, for the diagnosis and evaluation of the development of impaired visual function in children of school age. For example, it has been shown that the use of the Rarebit fovea test, coupled with standard visual acuity test when children start school, could increase the potential of evaluating the functional loss, for example in conditions such as juvenile macular degeneration, prior to funduscopically obvious macular changes [33]. Indeed, given that the reduction of foveal vision in these patients causes a reduction of oculomotor control with consequent decrease of fixation stability, adding Rarebit Fovea Test (not involving stable fixation) to visual acuity test (involving stable fixation) might provide a more precise evaluation of the visual deficit. Moreover, the Rarebit fovea test could be useful to establish whether in children with amblyopia the decrease of best corrected visual acuity is exclusively due to inhibition of normal visual pathway or also to the presence of high order aberration [45–46]. It has been questioned whether the comparison of foveal function between amblyopic and fellow eye in children with amblyopia (using Rarebit fovea test), could be used to diagnose the visual impairment [34]. The authors didn’t find any statistical difference between amblyopic and fellow eye. However, the use of *d’* instead of MHR as dependent variable could have been more appropriate, given that the number of response errors made during Rarebit testing was significantly higher in the amblyopic with respect to the fellow eye.

In normally sighted participants, the source of the difference in sensitivity, which we found to be remarkably independent of bias and task repetition, should be sought at all stage that occurs from stimulus presentation to response: stimulus representation mechanisms, higher-order brain mechanisms involved in the formation of a perceptual decision and the of use a wrong decision rule [28]. Age differences could be due to the inefficiency of stimulus representation mechanisms that result from either the physical randomness in the external environment (external noise) or the internal variability in the neural system [46]. We can exclude inefficiency of stimulus representation as a cause for two reasons: first, inefficient stimulus representation limits performance at a threshold level [26,47–48], whereas the group difference in *d’* was found with very large *d’*; second, Rarebit is not a thresholding perimetry. Stimuli are suprathreshold in normal vision [49]. Suprathreshold performance results from the use of target dots with high luminance presented on a very low luminance background, the use of a high number of stimulus repetitions (five) at each location and the long stimulus duration (200 ms) that positively relates to sensitivity [30,49]. The criterion is also unlikely to account for the age effect on *d’* because SDT states that *d’* is unaffected by response bias. Indeed, we found that the difference in sensitivity persists when there is no more difference in C among the two groups. The remaining cause for the reduction of suprathreshold sensitivity in children is the inefficient read-out of the sensory signals by the brain; signal representation needs to be interpreted (read-out) by higher order neural mechanisms to form a perceptual decision. Inefficient read-out of strong (suprathreshold) signals may result from different sources of extraneous noise, such as lapses of attention, reduced practice or a conflict with an irrelevant response, and are reflected in reduced asymptotic lapse rate due to false negative response (where p(false negative) = p(misses) = 1—p(MHR)) [50]. Specifically, a false negative indicates that, because of extraneous noise factors such location uncertainty, lapse of attention, insufficient task experience [47], the observer makes the wrong response (misses) even if the stimulus strength is above threshold. As Frisen [15] pointed out, Rarebit is not immune to a false negative; our measurement returns, after task learning and independent of bias, a moderate percentage of false negatives in adults (5%) and moderately high percentage of false negatives in children (10%). The source of extraneous noise should be sought in post-sensory decision-making neural mechanisms. Missing a strong signal reflects the inability of the brain to appropriately read out the neural response to sensory information that is well represented by sensory mechanisms and manifest in a sensitivity change [26,28,48,51]. For example, Ling and Carrasco [51] demonstrate that the effect of transient attention was reflected by a “response gain model” because it manifested at asymptote performance where accuracy was approximatively 90%. They found that, whereas sustained attention affected threshold, transient attention affected both threshold and the asymptote of the psychometric function.

Casco and colleagues [26] measured the effects of aging on *d’* when discriminating the direction of orientation offset from the vertical. They found that high *d’* values obtained at large orientation offsets were reduced by aging, indicating that aging is associated with a difficulty of suprathreshold non-signal inhibition by decision making neural mechanisms.

We can assume that when Rarebit testing results in a group difference for medium-high values of *d’* not associated with a difference in C, this indicates, rather than inefficient representation of the strong (suprathreshold) stimulus by sensory mechanism, an inefficiency in the neural mechanism underlying decision making, possibly resulting from the fact that the task requires distributed attention over the visual field in order to produce the sequence of saccades at the appropriate amplitude and latency.

Whereas performance of normally sighted adults and normally sighted experienced observers approaches that of an “ideal observers” and is almost at ceiling, the performance of children is not, possibly because the random change in fixation reduces the engagement of distributed attention to the task. It is well known that the capacity to perform eye movements, an index of spatial attention efficiency, does not reach adult levels until adolescence. Ross, Radant, Hommer and Young [52] performed a cross-sectional study using saccadic eye movements to assess several aspects of visuospatial attention in normal children ages 8–15 years. Saccadic latency (a global measure of the ability to shift visuospatial attention), the ability to suppress extraneous saccades during fixation and the ability to inhibit task-provoked anticipatory saccades reach adult levels between 10 and 15 years. Dye and Bavelier [53] found that the time required for attentional resources to recover after being directed towards the identification of a first target decreases as children’s ages increase.

In conclusion, although Rarebit perimetry has been shown to be a repeatable and reliable visual field test when using MHR as a dependent variable [54], the present results suggest that normative data for the adult and pediatric population should be reanalyzed to obtain a more reliable, bias-free, test–retest independent measure of sensitivity. With a bias-free index of sensitivity (*d’*), children’s performance is reduced with respect to adult performance. We suggest that the reduction is not to be accounted for with the inefficiency of sensory representation but, rather, high-level neural mechanisms involved in decision making.

## Supporting information

S1 File Data (mean hit rate, *d*’ and C) were available downloading this file. (XLSX)
